# The bladder cancer immune micro-environment in the context of response to immune checkpoint inhibition

**DOI:** 10.3389/fimmu.2023.1235884

**Published:** 2023-08-31

**Authors:** Jeroen van Dorp, Michiel S. van der Heijden

**Affiliations:** ^1^ Department of Molecular Carcinogenesis, Netherlands Cancer Institute, Amsterdam, Netherlands; ^2^ Department of Medical Oncology, Netherlands Cancer Institute, Amsterdam, Netherlands

**Keywords:** bladder cancer, immune checkpoint inhibition, immune micro-environment, tertiary lymphoid structure, PD-1, PD-L1, CTLA-4

## Abstract

Treatment with neoadjuvant cisplatin-based chemotherapy followed by radical cystectomy is the default treatment for muscle-invasive bladder cancer (BC). However, with the encouraging results of immune checkpoint inhibitiors (ICI) directed against PD-1/PD-L1 and CTLA-4 in recent years, the treatment landscape of BC is rapidly changing. In addition, it is becoming clear that the effect of ICI is highly dependent on the interaction between tumor cells and the tumor immune micro-environment (TIME). Different immune cells are involved in an anti-tumor response in BC. Cytotoxic CD8^+^ T-cells are the main effector cells, aided by other immune cells including other T-cells, B-cells and pro-inflammatory macrophages. As part of the ongoing anti-tumor immune response, lymphocytes aggregate in clusters called tertiary lymphoid structures (TLS). Tumor mutational burden (TMB) and infiltration of immune cells into the tumor are both important factors for establishing an anti-tumor immune response. In contrast, transforming growth factor beta (TGF-β) signaling in cancer-associated fibroblasts (CAFs) prevents infiltration of lymphocytes and potentially has an immunosuppressive effect. In conclusion, the effect of ICI seems to be reliant on a combination of tumor-intrinsic and TIME-related parameters. More research is needed to fully understand the underlying biological mechanisms to further improve patient care.

## Introduction

Urothelial carcinoma of the bladder, more commonly described as bladder cancer (BC), is the 10^th^ most common cancer worldwide (1, 2). In approximately 25% of BC cases, patients present with muscle-invasive disease, characterized by invasion of tumor cells into the muscularis propria layer of the urothelium. Standard treatment for muscle-invasive bladder cancer (MIBC) consists of radical cystectomy (RC) including removal of the locoregional lymph nodes (3–5). However, overall survival (OS) and recurrence-free survival (RFS) after surgery alone are still poor (6, 7).

Pre-treating patients with neoadjuvant cisplatin-based chemotherapy (NAC) leads to a pathological complete response (pCR) rate of 20-40% after RC (8). OS is improved by 5-8% compared to patients treated with a direct cystectomy with most benefit for patients that have a pCR after NAC (9).

Treatment with NAC has been the default treatment for MIBC for many years. However, this might change given the encouraging clinical efficacy of immune checkpoint inhibitors (ICI). It is becoming apparent that treatment with ICI is not a *one size fits all* treatment, but its efficacy is instead highly dependent on characteristics of the tumor and the tumor immune micro-environment (TIME). Here, we will review the research on ICI in MIBC and its reciprocal effects on the TIME.

## Immune checkpoint inhibitors

The physiological role of immune checkpoints is to maintain self-tolerance and regulate the extent and duration of inflammatory processes. However, these pathways are used by tumors in an attempt to escape from an inevitable immune response (10, 11). The mechanism of action of ICI is complex and dependent on pre-existing factors in the TIME such as the abundance and activation state of CD8^+^ T-cells, the presence of other immune cells and local cytokine signaling. In addition, treatment with ICI directly influences and changes the TIME, resulting in a delicate interplay. The best-known immune checkpoints are cytotoxic T-lymphocyte-associated protein 4 (CTLA-4) and programmed cell death protein 1 (PD-1) together with its ligands programmed death-ligand 1 and 2 (PD-L1 and PD-L2). However, more immune checkpoints are currently being investigated, including TIGIT, TIM3 and LAG3 (12).

CTLA-4 is expressed by T-cells, where it competes with CD28 for binding with CD80 or CD86, expressed primarily by antigen-presenting cells. Binding with CD28 elicits a signaling cascade eventually leading to T-cell activation (11, 13). Instead, binding of CTLA-4 to CD80 or CD86 results in an inhibitory response. By blocking the CTLA-4 receptor with monoclonal antibodies such as ipilimumab and tremelimumab, this inhibitory response can be negated, allowing for binding of CD80 or CD86 with CD28 to co-stimulate T-cell activation (11, 14). Ipilimumab and tremelimumab have been used in numerous clinical trials as monotherapy and in combination with other ICI. Both ipilimumab and tremelimumab have been approved by the U.S. Food and Drug Administration (FDA) and the European Medicines Agency (EMA) for clinical use, however neither drug have been approved as a standard treatment option for urothelial cancer specifically.

PD-1 is expressed by T-cells during initial antigen-mediated activation (15). By engaging its ligands PD-L1 and PD-L2, it functions to counter the activating signals after antigen stimulation, contributing to self-tolerance under physiological conditions (16–18). The PD-1 pathway is commonly exploited by tumor cells in order to evade the immune system. By inhibiting this pathway with monoclonal antibodies, an effective immune-mediated anti-tumor response can be mounted (19, 20). Multiple monoclonal inhibitory antibodies have been developed that target either PD-1 (nivolumab, pembrolizumab, cemiplimab and dostarlimab) or PD-L1 (durvalumab, avelumab and atezolizumab). These antibodies have been tested elaborately in multiple cancer types, including urothelial cancer, and are all approved by the FDA and EMA for clinical use.

## Immune checkpoint inhibition in the clinic

Standard first-line therapy for metastatic urothelial cancer (mUC) is cisplatin-based combination chemotherapy (21, 22). Carboplatin can be considered as an alternative for patients ineligible for cisplatin (21, 22). Following the success in other cancer types, such as melanoma, renal cell carcinoma and non-small cell lung cancer (NSCLC), ICI were tested in multiple trials in platinum-refractory mUC (**Table 1**) (23, 24). An improved OS was observed for pembrolizumab compared to chemotherapy in platinum-refractory mUC in the KEYNOTE-045 trial (23). These results led to the approval of pembrolizumab for the treatment of platinum-refractory mUC. The IMvigor211 trial explored atezolizumab as second-line therapy in mUC and showed improved OS for the intention-to-treat (ITT) population. However, this trial did not meet its primary endpoint of improved OS in patients with tumors with high PD-L1 expression (24).

**Table 1:** Overview of clinical trials assessing immune checkpoint inhibitors in bladder cancer.

Trial reference (ClinicalTrials.gov ID)	Trial title	Therapeutic antibody used	Primary findings	Reference
KEYNOTE-045 (NCT02256436)	A Study of Pembrolizumab (MK-3475) Versus Paclitaxel, Docetaxel, or Vinflunine for Participants With Advanced Urothelial Cancer (MK-3475-045/KEYNOTE-045)	Pembrolizumab	Pembrolizumab was associated with significantly longer OS (by approximately 3 months) than chemotherapy as second-line therapy for platinum-refractory mUC.	(23)
IMvigor211 (NCT02302807)	A Study of Atezolizumab Compared With Chemotherapy in Participants With Locally Advanced or Metastatic Urothelial Bladder Cancer [IMvigor211]	Atezolizumab	Atezolizumab was not associated with significantly longer OS than chemotherapy in patients with platinum-refractory mUC overexpressing PD-L1 (IC2/3).	(24)
KEYNOTE-361 (NCT02853305)	Study of Pembrolizumab With or Without Platinum-based Combination Chemotherapy Versus Chemotherapy Alone in Urothelial Carcinoma (MK-3475-361/KEYNOTE-361)	Pembrolizumab	The addition of pembrolizumab to first-line platinum-based chemotherapy did not significantly improve efficacy and should not be widely adopted for treatment of advanced urothelial carcinoma.	(25)
DANUBE (NCT02516241)	Study of MEDI4736 (Durvalumab) With or Without Tremelimumab Versus Standard of Care Chemotherapy in Urothelial Cancer	Durvalumab with or without tremelimumab	This study did not meet either of its coprimary endpoints of OS compared between the durvalumab monotherapy versus chemotherapy groups in the population of patients with high PD-L1 expression and between the durvalumab plus tremelimumab versus chemotherapy groups in the ITT population.	(26)
IMvigor130 (NCT02807636)	Study of Atezolizumab as Monotherapy and in Combination With Platinum-Based Chemotherapy in Participants With Untreated Locally Advanced or Metastatic Urothelial Carcinoma (IMvigor130)	Atezolizumab	Addition of atezolizumab to platinum-based chemotherapy as first-line treatment prolonged PFS in patients with mUC. The final OS analysis showed a non-statistically significant OS benefit (HR 0.85, p=0.023). OS for atezolizumab monotherapy vs chemotherapy was negative for the ITT population. An exploratory analysis showed a benefit for atezolizumab monotherapy in the PD-L1–high (IC2/3) group.	(27) ASCO-GU 2023
CheckMate-901 (NCT03036098)	Study of Nivolumab in Combination With Ipilimumab or Standard of Care Chemotherapy Compared to the Standard of Care Chemotherapy Alone in Treatment of Participants With Untreated Inoperable or Metastatic Urothelial Cancer (CheckMate901)	Nivolumab with or without ipilimumab	(2022): Nivolumab plus ipilimumab did not meet the primary endpoint of improved overall survival (OS) in patients with tumors with high PD-L1 expression; (2023): nivolumab in combination with cisplatin-based chemotherapy followed by nivolumab monotherapy demonstrated statistically significant benefits in OS and PFS.	2022/2023 BMS press release
CheckMate-274 (NCT02632409)	An Investigational Immuno-therapy Study of Nivolumab, Compared to Placebo, in Patients With Bladder or Upper Urinary Tract Cancer, Following Surgery to Remove the Cancer (CheckMate 274)	Nivolumab	Disease-free survival was longer with adjuvant nivolumab than with placebo in the ITT population and among patients with a PD-L1 expression level of 1% or more in patients with high-risk muscle-invasive urothelial carcinoma who were treated with radical surgery.	(28)
IMvigor010 (NCT02450331)	A Study of Atezolizumab Versus Observation as Adjuvant Therapy in Participants With High-Risk Muscle-Invasive Urothelial Carcinoma (UC) After Surgical Resection (IMvigor010)	Atezolizumab	The trial did not meet its primary endpoint of improved disease-free survival in patients receiving adjuvant atezolizumab over observation.	(29)
JAVELIN Bladder 100 (NCT02603432)	A Study Of Avelumab In Patients With Locally Advanced Or Metastatic Urothelial Cancer (JAVELIN Bladder 100)	Avelumab	Maintenance avelumab plus best supportive care significantly prolonged OS, as compared with best supportive care alone, among patients with urothelial cancer who had disease that had not progressed with first-line chemotherapy.	(30)
PURE-01 (NCT02736266)	Neoadjuvant Pembrolizumab for Muscle-invasive Urothelial Bladder Carcinoma	Pembrolizumab	Neoadjuvant pembrolizumab resulted in 42% of patients with pT0 and was safely administered in patients with MIBC.	(31)
ABACUS (NCT02662309)	Preoperative MPDL3280A in Transitional Cell Carcinoma of the Bladder (ABACUS)	Atezolizumab	The pCR rate was 31% (95% confidence interval: 21–41%), achieving the primary efficacy endpoint.	(32)
Preoperative durvalumab and tremelimumab (NCT02812420)	Durvalumab and Tremelimumab in Treating Patients With Muscle-Invasive, High-Risk Urothelial Cancer That Cannot Be Treated With Cisplatin-Based Therapy Before Surgery	Durvalumab with tremelimumab	The primary endpoint was safety and we observed 6 of 28 patients (21%) with grade ≥3 immune-related adverse events. We also observed pathological complete response of 37.5% of patients who completed surgery (n = 24).	(33)
NABUCCO (NCT03387761)	Neo-Adjuvant Bladder Urothelial Carcinoma COmbination-immunotherapy (NABUCCO)	Nivolumab with ipilimumab	All patients were evaluable for the study endpoints and underwent resection, 23 (96%) within 12 weeks (primary endpoint; feasibility). Grade 3-4 immune-related adverse events occurred in 55% of patients. Eleven patients (46%) had a pCR, meeting the secondary efficacy endpoint.	(34)
NABUCCO 2 (NCT03387761)	Neo-Adjuvant Bladder Urothelial Carcinoma COmbination-immunotherapy (NABUCCO)	Nivolumab with ipilimumab	A pCR was observed in six (43%) patients in cohort 2A (ipi 3 mg/kg) and in one (7%) patient in cohort 2B (ipi 1 mg/kg). Absence of plasma ctDNA correlated with pCR.	(35)
CheckMate-032 (NCT01928394)	A Study of Nivolumab by Itself or Nivolumab Combined With Ipilimumab in Patients With Advanced or Metastatic Solid Tumors	Nivolumab with or without ipilimumab	Objective response rate was 25.6%, 26.9%, and 38.0% in the NIVO3, NIVO3+IPI1, and NIVO1+IPI3 arms, respectively. Grade 3 or 4 treatment-related adverse events occurred in 21 (26.9%), 32 (30.8%), and 36 (39.1%) patients treated with NIVO3, NIVO3+IPI1, and NIVO1+IPI3, respectively.	(36)
EV-101 (NCT02091999)	A Study of Escalating Doses of ASG-22CE Given as Monotherapy in Subjects With Metastatic Urothelial Cancer and Other Malignant Solid Tumors That Express Nectin-4	Enfortumab Vedotin	Single-agent EV was generally well tolerated and provided clinically meaningful and durable responses in patients with mUC.	(37)
EV-301 (NCT03474107)	A Study to Evaluate Enfortumab Vedotin Versus (vs) Chemotherapy in Subjects With Previously Treated Locally Advanced or Metastatic Urothelial Cancer (EV-301)	Enfortumab Vedotin	Enfortumab vedotin significantly prolonged survival as compared with standard chemotherapy in patients with locally advanced or mUC who had previously received platinum-based treatment and a PD-1 or PD-L1 inhibitor.	(38)
EV-103 (NCT03288545)	A Study of Enfortumab Vedotin Alone or With Other Therapies for Treatment of Urothelial Cancer (EV-103)	Enfortumab Vedotin and pembrolizumab	Enfortumab vedotin plus pembrolizumab showed a manageable safety profile and promising confirmed objective response rate in cisplatin-ineligible pts with locally advanced or mUC; activity was consistently observed across a range of pre-specified subgroups including those with poor prognosis.	(39, 40)
EV-302 (NCT04223856)	Enfortumab Vedotin and Pembrolizumab vs. Chemotherapy Alone in Untreated Locally Advanced or Metastatic Urothelial Cancer (EV-302)	Enfortumab Vedotin and pembrolizumab	Pending	(41)

Encouraged by these first results of ICI, a number of phase 3 trials were initiated to investigate the effect of first-line treatment with anti-PD-1/PD-L1 compared to standard platinum-based chemotherapy (**Table 1**). These included the KEYNOTE-361 (pembrolizumab), DANUBE (durvalumab) and the IMvigor130 (atezolizumab) trials. Unfortunately, no meaningful improvement in clinical benefit for anti-PD-1/PD-L1 monotherapy versus standard first-line platinum-based chemotherapy treatment was observed (25, 26, 42). Both the KEYNOTE-361 and IMvigor130 trials also tested the effect of combined chemotherapy and ICI in this setting. Comparable to treatment with ICI monotherapy, the combination treatment did not confer any benefit over standard treatment with chemotherapy in the ITT analysis (25, 27). The results of another phase 3 trial (CheckMate-901) are still pending. In this trial the investigators assessed the effect of standard cisplatin-based chemotherapy together with nivolumab. A 2023 press release reported a statistically significant improvement in progression-free survival (PFS) and OS for cisplatin/gemcitabine plus nivolumab, in comparison with cisplatin/gemcitabine alone. Interestingly, a subgroup analysis of the IMvigor130 study suggested that the combination of cisplatin-based chemotherapy with atezolizumab had better synergy than the carboplatin-based combination (27), potentially explaining this phenomenon. The full results of the CheckMate-901 trial are pending and are required to better understand the discrepancies between these results and the results from the other first-line trials with ICI.

The DANUBE (durvalumab plus tremelimumab) and CheckMate-901 (ipilimumab plus nivolumab) trials also evaluated the combination of PD-1/PD-L1 inhibition with CTLA-4 inhibition in mUC (26, 42). The DANUBE trial did not reach its primary endpoint(s). However, a numerical difference can be observed in favor of combined treatment with durvalumab plus tremelimumab compared to durvalumab monotherapy, especially in the PD-L1^+^ population (26). The formal results of the CheckMate-901 trial are still pending, however, the trial failed to meet one of its (co)primary endpoints of improved OS for ipilimumab plus nivolumab in patients with tumors with high PD-L1 expression, according to a 2022 press release by Bristol Myers Squibb.

In defining the optimal treatment setting, some recent trials suggest there is a place for ICI in the adjuvant setting in MIBC (**Table 1**). Two phase 3 trials have explored the efficacy and clinical benefit of adjuvant nivolumab (CheckMate-274) and atezolizumab (IMvigor010) in patients with residual muscle-invasive disease after RC (28, 29). Only the trial with nivolumab met its primary endpoint of improved RFS in the ITT population, as well as in the PD-L1^high^ population (28). However, as PD-1/PD-L1 inhibition is approved as second-line treatment for mUC, it is possible that the group that did not receive adjuvant nivolumab will eventually benefit from checkpoint blockade in a later disease stage, as the disease would still be naïve to exposure to checkpoint inhibition. Therefore, OS data are needed to fully assess clinical benefit.

Interestingly, clinical benefit in the CheckMate-274 trial was most prominent in patients that were previously treated with NAC. A potentially similar phenomenon has also been observed in the phase 3 JAVELIN Bladder 100 trial, where patients were treated with avelumab as maintenance therapy after platinum-based treatment in the metastatic setting (30). Patients treated with avelumab had an improved OS compared to the group receiving best supportive care (30). In addition to trials investigating the feasibility in the adjuvant or metastatic setting or as maintenance therapy, a number of phase 1/2 trials investigated ICI in the neoadjuvant setting, primarily to offer an alternative for patients who refused cisplatin or were ineligible (**Table 1**). Preoperative pembrolizumab was investigated in the PURE-01 trial (31, 43). Patients with cT2-4N0 BC were treated with three cycles of pembrolizumab, followed by RC. A pCR rate of 38.5% was observed (31, 43). In the ABACUS trial, preoperative atezolizumab was investigated in cT2-4aN0 BC patients (32). This trial yielded a response rate of 31% (32).

Following up on the success of these trials, two neoadjuvant trials were initiated using a combination of CTLA-4 inhibition and PD-1/PD-L1 inhibition (33, 34). In the NABUCCO trial, 24 patients with locally advanced BC were treated with a combination of ipilimumab plus nivolumab followed by radical surgery (34). Here, a pCR was observed in 46% of patients. In addition, 58% of patients had no remaining residual muscle-invasive disease after surgery (ypT0/Tis/Ta/TaN0) (34).

The group of Gao and colleagues conducted a trial with two cycles of tremelimumab plus durvalumab in locally advanced BC. A pCR was observed in 38% of patients that underwent surgery, which is comparable to platinum-based chemotherapy regimens in locally advanced disease (8, 33).

In addition to the results observed in the NABUCCO trial, encouraging results with preoperative ipilimumab plus nivolumab have been observed in multiple other tumor types (44–48). These studies suggested a lower dose of ipilimumab may be sufficient in the non-metastatic setting. To find the optimal dose of ipilimumab and nivolumab in locally advanced BC, patients in cohort 2 of the NABUCCO trial were randomized to receive either two cycles of 3 mg/kg ipilimumab plus 1 mg/kg of nivolumab or two cycles of 1 mg/kg ipilimumab plus 3 mg/kg of nivolumab in both arms followed by one cycle of 3 mg/kg nivolumab and RC (35). A pCR was observed in 43% of patients treated with ipilimumab 3 mg/kg in combination with nivolumab, similar to the results from cohort 1 of the NABUCCO trial. In contrast, a pCR was observed in only 7% of patients treated with ipilimumab 1 mg/kg in combination with nivolumab (35).

Similarly, in the CheckMate-032 trial, patients with advanced BC were treated with either nivolumab monotherapy, or in combination with ipilimumab with different dose combinations (36, 49). While this trial was not properly powered to detect a difference in OS, a higher objective response rate was observed for patients treated with plus ipilimumab 3 mg/kg plus nivolumab 1 mg/kg (38.0%) compared to patients treated with ipilimumab 1 mg/kg plus nivolumab 3 mg/kg (26.9%) or nivolumab monotherapy (25.6%) (49).

Taken together, the data in BC suggests that a high dose of CTLA-4 blockade in combination with PD-1/PD-L1 blockade yields better clinical responses compared to a low dose of CTLA-4 blockade. For the locally advanced setting, this could be an alternative treatment especially for cisplatin-ineligible patients.

## The tumor immune micro-environment in bladder cancer

Across different tumor types, there are certain aspects that impact the general immunogenicity of tumors and the general efficacy of ICI. Tumor mutational burden (TMB) is a metric to indicate the average number of mutations in the DNA of tumor cells compared to healthy cells. Only a small fraction of these mutations are ‘driver’ mutations, while the majority are ‘passenger’ mutations with no direct function in tumor development or progression (50, 51). Potentially, these ‘passenger mutations’ generate aberrant proteins which can be detected by the immune system as neoantigens, triggering an immune response directed against the tumor (52, 53). TMB is a surrogate measure of neoantigen load, which allows it to serve as a predictive biomarker for general immunogenicity and tendency of tumors to respond to ICI (54–56). TMB varies per individual tumor. However, different tumor types have a different average TMB. Melanoma and other skin cancers typically have the highest TMB. Although not as high as melanoma, average TMB in BC is relatively high, similar to NSCLC (57, 58).

TMB has been investigated in a number of BC trials mentioned earlier. In the preoperative setting, it was positively associated with response in the PURE-01 trial (pembrolizumab), and numerically higher in responders compared to non-responders in the ABACUS trial (atezolizumab), NABUCCO trial (ipilimumab plus nivolumab) and in the preoperative trial with tremelimumab and durvalumab (31–34). In addition, changes in the TMB after treatment were assessed in the PURE-01 trial (pembrolizumab) in fourteen patients for which paired tissue samples were available (≥ypT2). Interestingly, TMB was significantly lower compared with the baseline TMB after treatment with pembrolizumab (31).

In addition to an increased TMB, some specific genomic alterations also impact tumor behavior, prognosis and response to ICI. Recently, it was shown that loss of the Y-chromosome is associated with poor prognosis in male BC patients and was related to intratumoral CD8^+^ T-cell dysfunction and exhaustion. Interestingly, patients with loss of the Y-chromosome exhibited an increased response to PD-1 inhibition in both mice and BC patients (59).

Multiple immune cell subsets are implied to play a role in the TIME. Cytotoxic CD8^+^ T-cells have been established as one of the major players in the TIME in BC as well as in most other tumor types. Within the CD8^+^ T-cells, different subsets have been observed with varying degrees of tumor-reactivity. CD8^+^ T-cells expressing the combination of CD103 (integrin αE) and CD39 (an ectonucleotidase) are enriched for tumor-reactive cells in multiple different tumor types. These cells also efficiently kill autologous tumor cells in a major histocompatibility complex (MHC) class I-dependent manner (60). Specifically in BC, it has been shown that patients with tumors with high infiltration of CD8^+^CD103^+^ tissue-resident memory T-cells are more likely to benefit from ICI and adjuvant chemotherapy (61).

Based on the abundance of CD8^+^ T-cells and other immune cells and their spatial organization in relation to the tumor, distinct immune phenotypes can be defined (62, 63).

*Immune-Inflamed* tumors are considered to be immunologically ‘hot’ tumors and are characterized by an abundancy of immune cells invading the tumor and the surrounding stroma. Apart from CD8^+^ T-cells, these include other T-cells, B-cells and pro-inflammatory macrophages. In addition, these tumors are characterized by a type I Interferon gamma (IFN-γ) signature (63).

Tumors that are populated with immune cells but with relatively few cytotoxic T-cells inside the core of the tumor are commonly referred to as tumors with an *immune-excluded* phenotype (62, 63). It is currently not completely understood if these cytotoxic cells are insufficiently stimulated to infiltrate the tumor, or whether tumor infiltration is physically being prevented by interfering fibroblasts or stromal cells or due to other pro-tumorigenic cells (64). It has been suggested that tumor-associated macrophages along the tumor margins prevent cytotoxic lymphocytes from tumor core infiltration (65).

Tumors with very few immune cells are referred to as immunologically ‘cold’ tumors or having an *immune-desert* phenotype. These tumors are characterized by a low number of lymphocytes and a high macrophage-to-lymphocyte ratio (66).

Multiple studies have shown that greater infiltration of CD8^+^ T-cells is related to a more favorable clinical outcome and a better response to ICI in multiple disease stages in BC (32, 67–71).

In the ABACUS trial mentioned above, a relatively high proportion of immune-inflamed (73%) tumors was found based on the abundance and spatial organization of CD8^+^ T-cells. However, the immune-inflamed phenotype did not correlate with response (32). A CD8/GZMB co-staining was performed to further enrich for tumors with high anti-tumor reactivity. Indeed, the percentage of tumors with an immune-inflamed phenotype that contained CD8^+^GZMB^+^ cells was higher in responders versus patients that relapsed (87% and 30%, respectively) (32). In addition, a significant increase in infiltrating CD8^+^ T-cells was observed based on immunohistochemistry when comparing post-treatment tissue to pre-treatment tissue (32).

In the PURE-01 trial mentioned above, the effect of ICI on the TIME was assessed by comparing pre- and post- treatment samples from patients with residual disease after treatment with preoperative pembrolizumab (72). These findings were compared to patients that were treated with a direct RC, or with NAC followed by RC. It was found that patients with residual tumor after treatment with pembrolizumab and cystectomy showed a high rate of stroma-rich calls with a decreased tumor purity and increased stromal content (72). In addition, these tumors also expressed luminal markers, distinguishing them from the untreated and tumors that did not respond to NAC. This would suggest that luminal tumors may have an intrinsic resistance to treatment with ICI or that treatment with ICI may select for, or induce, a luminal phenotype (72, 73)

In another study, immune phenotypes were classified based on CD8^+^ T-cell density in the tumor and stroma compartments in an untreated BC cohort (74). Immune-inflamed (42%) was the most common immune phenotype, whereas 32% and 26% of tumors were classified as immune-excluded and immune-desert phenotypes, respectively. Although tumors qualified as immune-desert showed a numerically high rate of recurrence (88%), no statistically significant correlation was found (74).

In contrast, in the NABUCCO trial (preoperative ipilimumab plus nivolumab) no correlation was observed between baseline CD8^+^ T-cell density and response to ipilimumab plus nivolumab. In addition, no significant difference was observed in IFN-γ signaling at baseline in responding tumors compared to non-responding tumors. This data suggests that the addition of anti-CTLA-4 to PD-1 blockade can induce a pCR in tumors irrespective of baseline immunity (34). In addition, the density of CD8^+^PD1^+^ T-cells in tumors from patients treated with ipilimumab and nivolumab was higher than that of patients treated with a direct cystectomy, regardless of response to ICI (74).

Current efforts to understand anti-tumor immunity are primarily focused on CD8^+^ T-cells. However, there is also a role for CD4^+^ T-cells in the interaction between the tumor and TIME. Regulatory CD4^+^ T-cells in the BC TIME are known for their role in inhibiting or dampening an ongoing immune response by producing anti-inflammatory cytokines like IL-10 and transforming growth factor beta (TGF-β), direct inhibition of dendritic cells and more (75, 76). Regulatory CD4^+^ T-cells in the BC TIME have been associated with adverse outcomes, similar to other tumor types (77). The exact underlying biological mechanism for this association remains unclear. However, one study found overexpression of sphingosine 1 phosphate receptor 1 (S1P1) in BC, promoting production of TGF-β and IL-10 *in vitro* and *in vivo* (78).

In addition to an immunosuppressive role, it was recently found that CD4^+^ T-cells also play an important role in anti-tumor immunity in BC (79). Based on data from single-cell RNA sequencing, multiple cytotoxic CD4^+^ T-cell states were identified based on the expression of granzyme B, granzyme K, perforin as well as other granule-associated proteins. These distinct populations were validated by flow cytometry and multiplex immunofluorescence tissue staining. In addition, it was found that cytotoxic CD4^+^ subsets in bladder tumors were clonally expanded, potentially resulting from recognition of bladder tumor antigens. Their functional importance was confirmed by their ability to kill autologous tumors *ex vivo* in a MHC class II-dependent manner. Overall, these findings highlight the importance of CD4^+^ T-cell heterogeneity and the relative balance between activation of cytotoxic CD4^+^ effector cells and inhibitory regulatory cells for killing autologous tumors (79).

Apart from lymphocytes, there is a prominent role for macrophages in the TIME. Macrophages are not a single cell population with a defined phenotype and biological activity but rather a diverse collection of cell types with a wide range of functional roles (80). Macrophages have been traditionally categorized as either M1 or M2 macrophages, characterized by different markers. M1 macrophages are considered anti-tumorigenic and express high levels of tumor necrosis factor alpha (TNFα), inducible nitric oxide synthase (iNOS) or MHC class II molecules. In contrast, M2 macrophages are considered pro-tumorigenic and express CD163, CD206 and high levels of arginase 1 (ARG1) and IL-10 (81). However, it is becoming clear that a broad spectrum of macrophage phenotypes exists, and using markers to delineate their functional role within the tumor is not straightforward (82). The majority of the work on macrophages has been done in other tumor types, but a few studies also investigated whether there is an association between macrophage abundance and polarization status (M1-like or M2-like) and prognosis and outcome in BC.

One study found that a high intratumoral density of M2 macrophages (based on expression of CD163) was associated with poor outcome in patients with BC (83). Another study by Sun and colleagues investigated macrophage polarization in relation to response to ICI treatment in the IMvigor210 trial (atezolizumab). It was found that patients with tumors with predominantly M1 macrophages were indeed more sensitive to PD-L1 blockade (84). Wang and collaborators found that a pro-tumorigenic inflammatory signature was correlated with poor outcome in the IMvigor210 (atezolizumab) and the CheckMate-275 (nivolumab) trials (85). One preclinical study used a conditional knockout BC mouse model which showed a heterogeneous response to treatment with PD-1 inhibition. Responding tumors showed a higher number of intratumoral macrophages (86).

While the exact mechanism still needs to be unraveled, the number of macrophages and their polarization status seems to be associated with the efficacy of ICI and thus may be relevant to predict which patients respond to treatment.

Similar to other tumor types, multiple other cell types likely also play a role in the TIME in BC. These include, but are not limited to: pericytes, dendritic cells, natural killer cells and eosinophils. While important, limited work has been done in BC specifically.

Tertiary lymphoid structures (TLS) are organized clusters of lymphocytes in chronically inflamed tissue (87). They resemble secondary lymphoid organs like lymph nodes and have similar functions such as mounting germinal center reactions and priming of antigen-specific T-cells (88, 89). The density of TLS in the TIME is associated with infiltration of adaptive immune cells and improved clinical outcome in multiple tumor types including in BC (90–92). However, it is currently unclear what the exact role is of TLS in the anti-tumor immune response and whether they are a prerequisite or rather a consequence of an anti-tumor immune response (91). It was proposed that TLS mature through different developmental stages with increasing proportions of activated lymphocytes throughout the maturation process (93). However, another study assessing different BC cohorts found that the proportions of activated B-cells, T-cells and progenitor-like CD8^+^ T-cells were similar when comparing maturation stages, and seemed to be more dependent on the number of TLS in the TIME (91).

Another study in BC made a distinction between superficial and deep TLS, based on their location relative to the bladder lumen. Superficial TLS were hypothesized to primarily play a role in the immune response against irritative chemicals and microbial pathogens present in the urine. Deep TLS presumably play a more prominent role in anti-tumor immunity. It was found that the density of CD4^+^ T-cells was higher in superficial TLS and the proportion of follicle-like, mature structures was higher in deep TLS (74).

Two trials that assessed combination ICI as a preoperative strategy in locally advanced BC investigated TLS at baseline as a predictive biomarker. One study testing preoperative tremelimumab plus durvalumab reported a higher density of pretreatment TLS in responders compared to non-responders (33). In addition, it was observed that a higher density of pretreatment TLS was associated with a longer OS and RFS.

In contrast, no difference in pretreatment TLS density was reported in responders compared to non-responders in patients with locally advanced BC treated with preoperative ipilimumab plus nivolumab in the NABUCCO trial (34). However, the density of TLS after treatment increased in responders, whereas the TLS density decreased in non-responders. In addition, it was found that regulatory T-cells in TLS decreased after treatment with ipilimumab plus nivolumab, showing that this treatment also influences the composition of TLS (34). In another recent study in BC, an association was found between TLS density and TMB as well as increased T-cell activation. Combining TLS density with TMB into a joint ‘TLSTMB’ score generated a novel prognostic biomarker that, in contrast to either TLS density of TMB alone, was independent from tumor stage and vascular invasion (91). The exact role of TLS remains to be elucidated. However, it is clear from multiple studies that TLS play a pivotal role in the anti-tumor immune response in BC.

**Figure 1:**
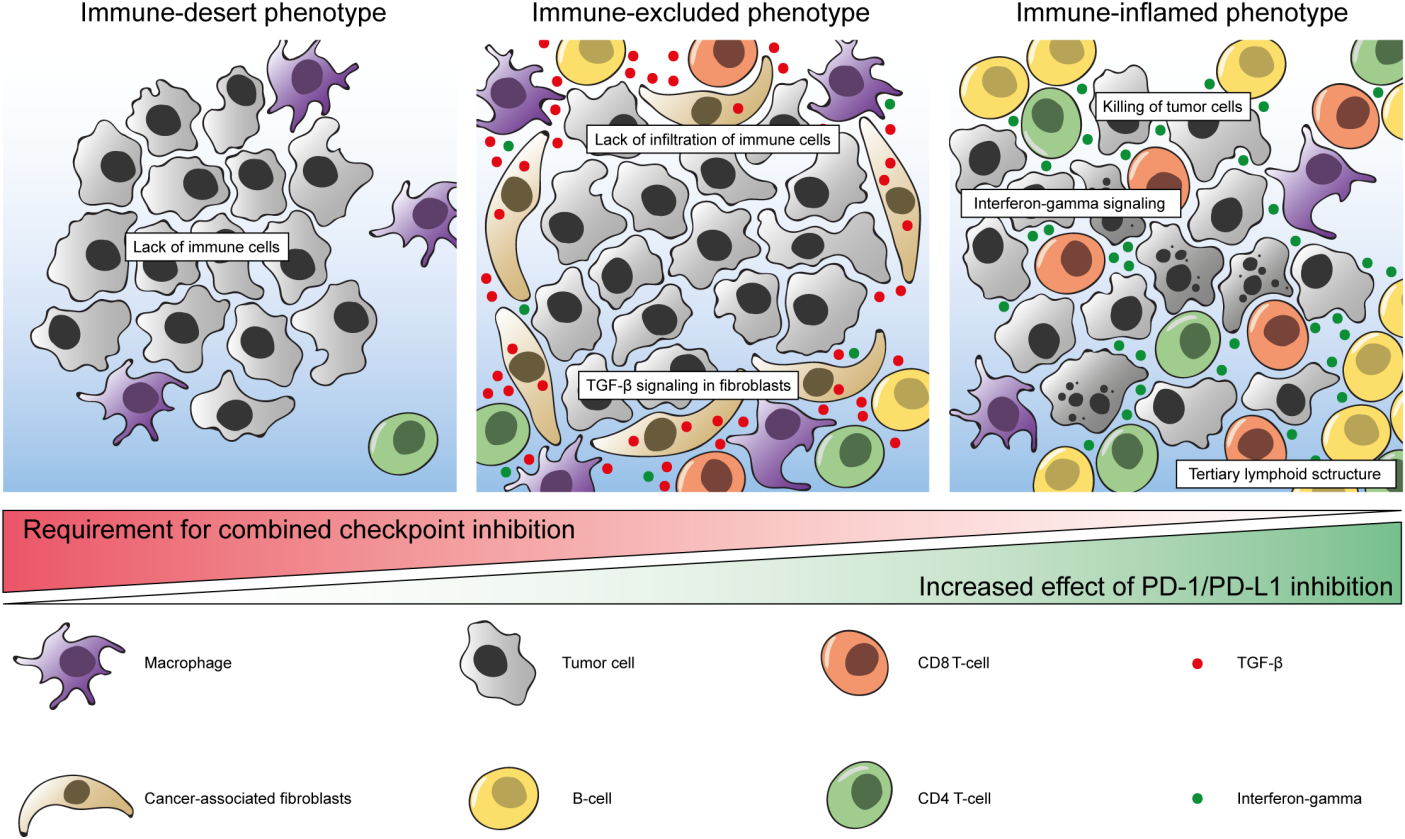
Model of immune phenotypes in the bladder tumor immune micro-environment. Left: immune-desert phenotype with limited amounts of immune cells. Middle: immune-excluded phenotype with stromal cells, cancer associated fibroblasts and TGF-β signaling preventing infiltration of CD8^+^ T-cells and other immune cells. Macrophages predominantly display an immunosuppressive (M2) phenotype. Right: immune-inflamed phenotype with extensive infiltration of CD8^+^ T-cells and other immune cells. Macrophages are primarily of the M1 phenotype. Bottom: Increased effect of PD-1/PD-L1 inhibition have been observed in tumors with an immune-inflamed phenotype. Addition of CTLA-4 inhibition might be required to mount an effective immune response in tumors without pre-existing immunity.

TGF-β is a cytokine that is involved in multiple different pathways and interactions and is associated with poor clinical outcome in different tumor types (94–96). It is thought to have a pro-tumorigenic role in advanced cancers by promoting fibroblast activation, immunosuppression, angiogenesis, epithelial-to-mesenchymal transition, and metastasis (97). In a study by Mariathasan and colleagues, it was found that expression of TGF-β ligand 1 (TGFB1) and TGF-β receptor 2 (TGFBR2) were associated with non-response and reduced OS in patients with mUC who were treated with atezolizumab in the IMvigor210 trial (71). In addition, the authors speculated that TGF-β signaling in cancer-associated fibroblasts (CAFs) contributed to an immune-excluded TIME. To measure TGF-β signaling specifically in fibroblasts, a pan-fibroblast TGF-β response signature (F-TBRS) was created. Expression of this signature was particularly high in tumors with an inflamed or excluded phenotype, and low in tumors with an immune desert phenotype. In line with these findings, the F-TBRS was significantly associated with non-response in excluded tumors specifically (**Figure 1**) (71). In addition, it was confirmed in an EMT6 mouse mammary carcinoma model that combining PD-L1 blockade and a TGF-β inhibitor led to infiltration of lymphocytes into the tumor and an improved survival, whereas this was not observed upon monotherapy with either inhibitor (71).

In the ABACUS trial, patients were treated with atezolizumab in the preoperative setting. Among the patients that relapsed, the patients with an immune-excluded bladder tumor had a numerically higher expression of the TGF-β response signature, which was not the case for patients that responded. However, no definitive conclusion can be drawn from this study due to lack of statistical power (32). In the NABUCCO trial (ipilimumab plus nivolumab), a significantly higher expression of the TGF-β response signature was observed in non-responders versus responders at baseline (34).

A number of TGF-β inhibitors have been developed for clinical use and are currently being investigated in early clinical trials (98). To our knowledge, there are currently two trials investigating TGF-β inhibitors in patients with mUC. In one trial, patients are treated with the oral TGF-β inhibitor vactosertib in combination with durvalumab (NCT04064190), for which results are pending. The other trial tested bintrafusp α (M7824), a bifunctional fusion protein composed of the extracellular domain of a TGF-β receptor fused to PD-L1 antibody (NCT04501094). This trial has been terminated due to low accrual.

## Modulating the tumor immune micro-environment to improve therapy response

In recent years, encouraging responses to ICI have been observed in BC patients. However, there is still a substantial subset of patients that does not respond to this treatment. This ICI resistance might be explained by various mechanisms, including tumor-intrinsic factors or factors related to the immune micro-environment (55, 56, 71). Combining ICI monotherapy with other drugs such as additional ICI or conventional chemotherapy, may alter the TIME to be more susceptible to respond to treatment.

Traditionally, chemotherapy has been regarded as immuno-suppressive, depleting immune cell subsets and leading to an increased rate of infections (99). However, it has been shown in numerous preclinical and clinical studies that treatment with chemotherapy can also have immunostimulatory effects (100, 101). One of the direct effects of chemotherapy is the induction of immunogenic cell death, a form of cell death that is being preceded by a cellular stress-response. Via complex intracellular pathways, phagocytosis of tumor cells (or portions thereof) by dendritic cells is facilitated (102). Processing of this cellular debris by dendritic cells eventually leads to presentation of neoantigens, as described earlier (52, 53).

In addition to the induction of immunogenic cell death, chemotherapy treatment also affects regulatory T-cells, tumor-associated macrophages and myeloid derived suppressor cells (103, 104). This phenomenon could be further exploited when used in conjunction with ICI. Indeed, a synergistic effect of concurrent chemotherapy and ICI has been observed in multiple tumor types, including in NSCLC and in triple-negative breast cancer (TNBC) (105–107).

In BC, one preclinical study assessed the effect of PD-L1 inhibition with or without platinum-based chemotherapy compared to platinum-based chemotherapy alone (108). The combination strategy was more effective in the MB49 subcutaneous model compared to either platinum-based chemotherapy or PD-L1 inhibition alone. Interestingly, PD-L1 inhibition monotherapy was more effective than the combination strategy in the MBT-2 subcutaneous model, suggesting that combined treatment results are model-dependent (108). Despite the positive results in other cancer types, no clinical benefit was observed when patients with mUC were treated with PD-1/PD-L1 inhibition or PD-1/PD-L1 inhibition in combination with standard platinum-based chemotherapy over chemotherapy alone in both the KEYNOTE-361 (pembrolizumab) and in the IMvigor130 (atezolizumab) trials (25, 27). However, a 2023 press release of the CheckMate-901 study reported a statistically significant improvement in PFS and OS for cisplatin/gemcitabine plus nivolumab, in comparison with cisplatin/gemcitabine alone. The full results are pending and are required to better understand the discrepancies between these trial results. Potentially, this might result in the first approved treatment strategy with concurrent chemotherapy and ICI in BC.

Theoretically, a sequential approach could be appealing, priming the TIME and allowing recovery of immune cell populations to subsequently further improve the anti-tumor response with ICI treatment (109). This sequential approach was studied in patients with TNBC, where patients were treated with different types of induction chemotherapy, followed by nivolumab (110). In metastatic BC, the JAVELIN Bladder 100 trial (avelumab) showed the efficacy of maintenance checkpoint inhibition after initial treatment with platinum-based chemotherapy (30). The improved results for adjuvant nivolumab in the subset of patients in the CheckMate-274 trial who received NAC similarly supports sequential treatment (28).

Given the accessibility of bladder tumors, the TIME could be modulated by local therapies to improve susceptibility to ICI treatment, without having to expose patients to systemic therapy. For example, intravesical instilments with Bacillus Calmette–Guerin (BCG) or chemotherapeutic agents such as epirubicin or mitomycin can be employed. BCG represents the first type of immunomodulatory treatment approved by the FDA (111). Despite its proven efficacy in reducing the chance of disease recurrence, its underlying biological mechanism is not fully understood. Generally, BCG is internalized primarily by cancer cells leading to cytokine production and the activation of CD4^+^ and CD8^+^ T-cells, leading to the killing of cancer cells (112, 113). Interestingly, one study showed that the efficacy of treatment with BCG is at least partly explained by PD-L1 expression, as the percentage of patients with PD-L1^+^ tumors at baseline was higher in patients that did not respond to treatment with BCG compared to patients that did respond (114). Indeed, systemic treatment with pembrolizumab in patients with BCG-unresponsive non-muscle invasive BC was tolerable and showed promising anti-tumor activity in a single-arm phase 2 trial, leading to the approval of pembrolizumab for BCG-unresponsive, high-risk non-muscle invasive BC (115).

Intravesical instilments with ICI could potentially evoke a local immune response with less systemic exposure. Two exploratory trials investigated whether intravesical instilments with pembrolizumab were feasible. In one trial, patients with BCG-unresponsive non-muscle invasive BC were treated with intravesical pembrolizumab. A significant increase in CD4^+^ T-cells and CD8^+^ T-cells was found in the urine after a single dose of pembrolizumab as well as an increase in infiltrating CD8^+^ T-cells in the tumor (116). Interestingly, even though treatment was only administered locally in the bladder, systemic immune-related adverse events were observed in some patients (116). In the PemBla trial, six patients were treated with increasing doses of intravesical installments of pembrolizumab after transurethral resection of the bladder, which was well tolerated. These two exploratory trials confirm prior pre-clinical observations in a mouse BC model (MBT-2), where intravesical PD-1 inhibition was used to treat localized BC, and showed a similar effect compared to systemic treatment with PD-1 inhibition and changes in the TIME including increased infiltration of CD8^+^ T-cells (117).

Enfortumab vedotin (EV) - an antibody-drug conjugate - is directed against nectin-4, a protein which is highly expressed in urothelial cancer cells and is linked to monomethyl auristatin E, an agent that disrupts microtubule formation (37, 118). Encouraging results have been observed when EV was used as monotherapy in pretreated mUC (**Table 1**) (38). In addition, it has also been observed that treatment with EV leads to hallmarks of immunogenic cell death leading to T-cell activation (118, 119). Combined with pembrolizumab, EV has shown promising results and this combination is currently under investigation in a phase 3 study (39–41).

## Conclusion

We have come to understand that cancer cells rely heavily on their interaction with the surrounding TIME, especially in the context of ICI. It is becoming clear which cell types, pathways and processes are involved in anti-tumor immunity. Taken together, a combination of tumor-intrinsic and microenvironment-related parameters determine the success of therapies targeting immune checkpoints: i) A high TMB and a high rate of neoantigens resulting in aberrant proteins which can be recognized by immune cells to then mount an effective antitumor immune response ii) Pre-existing anti-tumor immunity with infiltrating cytotoxic T-cells, IFN-γ signaling and the formation of TLS; iii) Low expression of TGF-β in CAFs to prevent an immune-excluded immune phenotype (**Figure 1**) (71, 120). However, despite meeting all of these criteria, some tumors still do not respond well to ICI. This indicates that there are still some missing pieces in the puzzle of adequate immunological cancer treatment.

While there are many commonalities across different tumor types, there are also some features related to the TIME that have been found specifically in BC. These include the importance of TGF-β signaling and role of CD4^+^ T-cells and might explain some of the unique clinical findings observed in BC studies. For example, the improved clinical outcome when a high dose of CTLA-4 inhibition is used together with PD1/PD-L1 inhibition and the apparent lack of synergy when treating with a combination of ICI and systemic chemotherapy.

Recent findings highlight the rapidly changing treatment landscape of BC. We now understand that cancer cell characteristics are just part of the puzzle for effective cancer treatment. Targeted therapeutic strategies like ICI and antibody-drug conjugates such as EV are highly dependent on the interaction between cancer cells and the TIME. Ultimately, more research is needed to better understand the TIME.

## Author contributions

JD and MH wrote the manuscript and created the illustrations. All authors contributed to the article and approved the submitted version.
